# Visible Light‐Activated ZnO@CuO Coaxial Nanofibers Enhance Infected Skin Wound Healing Through Construction of p‐n Heterojunctions

**DOI:** 10.1002/advs.202512700

**Published:** 2026-01-08

**Authors:** Pengrui Dang, Han Zhao, Chenguang Zhang, Jiechen Wang, Fangyu Zhu, Qinqiu Zhong, Wenyi Zeng, Xinyuan Wang, Yumin Chen, Xu Yan, Xuliang Deng, Wenwen Liu

**Affiliations:** ^1^ Department of Geriatric Dentistry Peking University School and Hospital of Stomatology & National Center for Stomatology & National Clinical Research Center for Oral Diseases & National Engineering Research Center of Oral Biomaterials and Digital Medical Devices & NMPA Key Laboratory For Dental Materials Beijing China; ^2^ The VIP Department Liaoning Provincial Key Laboratory of Oral Diseases School and Hospital of Stomatology China Medical University Shenyang China; ^3^ Department of General Dentistry Peking University School and Hospital of Stomatology & National Center for Stomatology & National Clinical Research Center for Oral Diseases & National Engineering Research Center of Oral Biomaterials and Digital Medical Devices & NMPA Key Laboratory For Dental Materials Beijing China; ^4^ Hospital of Stomatology Guanghua School of Stomatology Guangdong Provincial Key Laboratory of Stomatology Sun Yat‐sen University Guangzhou China; ^5^ Department of Stomatology Union Hospital Tongji Medical College Huazhong University of Science and Technology Wuhan China

**Keywords:** antibacterial dressing, coaxial nanofibers, infected wound healing, p‐n heterojunction, visible light photocatalysis

## Abstract

The noninvasive regulation of photocatalytic materials for reactive oxygen species (ROS)‐based antimicrobial strategies offers a promising approach for healing infected wounds under comorbid conditions. However, most ROS‐generating photocatalytic materials rely on ultraviolet (UV) or near‐infrared (NIR) light activation, presenting challenges for convenient application and biosafety. In this study, novel zinc oxide@copper oxide (ZnO@CuO) nanofibers with a robust coaxial structure are developed that could be activated by visible light to generate ROS through photocatalytic reactions. The orderly arrangement of the inner and outer semiconductor layers enhanced the contact efficiency of p‐n heterojunctions, thereby improving the electron‐hole separation efficiency in the nanofibers. The enhanced contact efficiency of p‐n heterojunctions is the key mechanism driving the improved photocatalytic properties and increased ROS generation under visible light, offering greater ease of application and biocompatibility. The ZnO@CuO coaxial nanofibers exhibited excellent antimicrobial properties and accelerated wound healing under visible light in a methicillin‐resistant Staphylococcus aureus (MRSA)‐infected mouse skin wound model. The coaxial nanofibers controlled infection at an early stage and significantly accelerated wound healing. Even in infected diabetic wounds, a healing rate of 92.3% is achieved within 10 days. This innovative approach utilizes biosafe visible light, providing a promising solution for infected wound therapies.

## Graphical abstract

Coaxial ZnO@CuO nanofibers activated by visible light generate ROS through enhanced photocatalytic reactions driven by improved electron hole separation in the heterojunction. This strategy enables rapid antimicrobial activity and early infection control, thereby promoting the healing of MRSA infected and diabetic wounds with biosafe and easily applicable visible light.

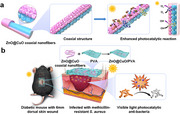

## 1 Introduction

Recurrently infected wounds have long been a major obstacle in clinical practice, particularly in patients with comorbid diseases, such as diabetes. Microvascular complications and immune function suppression caused by comorbid diseases often result in severe infections that can lead to amputation. This makes the accelerated healing of infected wounds even more challenging [1, 2]. Currently, antibiotics are the main treatment for infected wounds, but whether used locally or systemically, they carry the risk of drug resistance and low sustainability, delaying healing [3, 4]. Alternatively, various wound dressings incorporating metal ions, thermal effects, or reactive oxygen species (ROS) for their antimicrobial properties have been developed as additional approaches to promote wound healing [5, 6, 7, 8, 9]. Among these, ROS‐based antimicrobial strategies stand out for their ability to overcome biotoxicity and practical limitations commonly associated with other approaches [10, 11]. The development of wound dressings capable of generating ROS has therefore emerged as a promising solution for enhancing the healing of infected wounds [12, 13].

Photocatalytic materials have recently attracted attention for their ability to modulate ROS generation, providing benefits such as ease of application, noninvasiveness, and precise spatiotemporal control [14, 15]. Through the photocatalytic process, these dressings generate ROS to target infectious microorganisms in the wound microenvironment, promoting wound healing [16, 17]. The mechanism of action for regulating ROS concentration primarily involves two aspects. On one hand, during the proliferative phase of wound healing, an appropriate concentration of ROS not only promotes the recruitment of neutrophils and macrophages to the injured site to clear damaged tissue [18, 19], but also facilitates the production of collagen and other extracellular matrix (ECM) components. On the other hand, excessive accumulation of ROS may lead to oxidative stress, inhibiting wound healing [19, 20]. Notably, ROS storms are an effective strategy for killing or inhibiting bacteria in infected areas without inducing oxidative stress. Therefore, maintaining ROS at optimal levels is critical for local infection control and for accelerating the wound healing process. Most photocatalytic ROS‐generating materials rely on ultraviolet (UV) or near‐infrared (NIR) light [21, 22]. However, the biotoxicity of UV light limits its clinical use, and NIR‐responsive materials face high equipment costs and the risk of thermal damage to the surrounding healthy skin [23, 24]. In comparison, visible light is biologically safe, easily accessible, and free from adverse effects, making it an ideal choice for ROS generation via photocatalysis in antimicrobial applications [25, 26].

Most visible‐light‐driven photocatalytic materials rely on the construction of p‐n heterojunctions. These heterojunctions create an additional electric field that enhances photocatalytic performance by accelerating electron‐hole migration [27, 28, 29, 30]. Many of these heterojunctions incorporate nonbiocompatible metals or other biotoxic substances, making them unsuitable as raw materials for wound dressings [28, 29, 30]. Zinc oxide (ZnO) is a biocompatible n‐type semiconductor with excellent photocatalytic properties, whereas copper oxide (CuO), a p‐type semiconductor, can enable visible‐light photocatalysis by forming a p‐n heterojunction with ZnO [31]. The low contact efficiency between the p‐type and n‐type semiconductors in the existing ZnO/CuO heterojunction microstructures limits the separation of photogenerated electron‐hole pairs, thereby reducing the photocatalytic efficiency [32]. The coaxial structure, which is stable constructure in 1D nanomaterials, improves the contact efficiency of heterojunctions by facilitating an ordered arrangement of the inner and outer semiconductor layers [33]. Accordingly, ZnO/CuO heterojunctions with a coaxial structure show significant potential for enhancing antimicrobial properties by utilizing their superior photocatalytic ability to generate ROS, thereby promoting the healing of infected wounds through easy application under visible‐light‐responsive stimuli.

**FIGURE 1:**
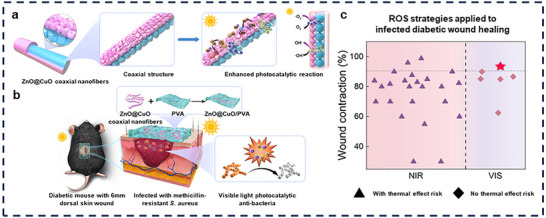
Design and working mechanism of ZnO@CuO coaxial nanofibers for efficient antimicrobial properties under visible light by optimizing photogenerated carrier dynamics and thus promoting infectious diabetic wound healing. (a) Mechanism of enhanced visible light photocatalytic performance by coaxial structures. (b) Scheme of ZnO@CuO/PVA for promoting infected diabetic wound healing. (c) Wound healing rates of ZnO@CuO/PVA (red pentagram) compared to other wound dressings under diabetic wound combined infection conditions. The materials were classified according to the excitation wavelength of light (purple for NIR, pink for VIS) and the presence or absence of a risk of thermal effect (▲ for with thermal effect risk, ◆ for no thermal effect risk). Wound healing rate is the ratio of the healed area to the initial wound area over days 7–10.

In this study, ZnO@CuO coaxial nanofibers were fabricated via coaxial electrospinning, which enabled photocatalytic reactions under visible light. These nanofibers featured biocompatible ZnO as the outer shell and CuO as the inner layer. This coaxial structure strengthens the internal electric field, improves the electron‐hole separation efficiency, and optimizes the photogenerated carrier dynamics of the heterojunctions (Figure 1a). The ZnO@CuO coaxial nanofibers exhibited superior photocatalytic performance. During photocatalytic reactions, ROS including hydroxyl and superoxide radicals are generated for antimicrobial purposes. This process enables rapid bactericidal clearance in the early stages of infection under easily accessible visible light‐responsive stimuli, thereby promoting healing of infected wounds (Figure 1b). For infected wound healing, especially under diabetic conditions, wound dressings loaded with visible‐light‐responsive ZnO@CuO coaxial nanofibers represent a promising strategy. It controls infection using easily applicable and biosafe visible light, achieving the highest wound contraction rate without causing adverse effects (Figure 1c).

## 2 Results and Discussion

### 2.1 Synthesis of ZnO@CuO Nanofibers with a Stable Coaxial Structure

**FIGURE 2:**
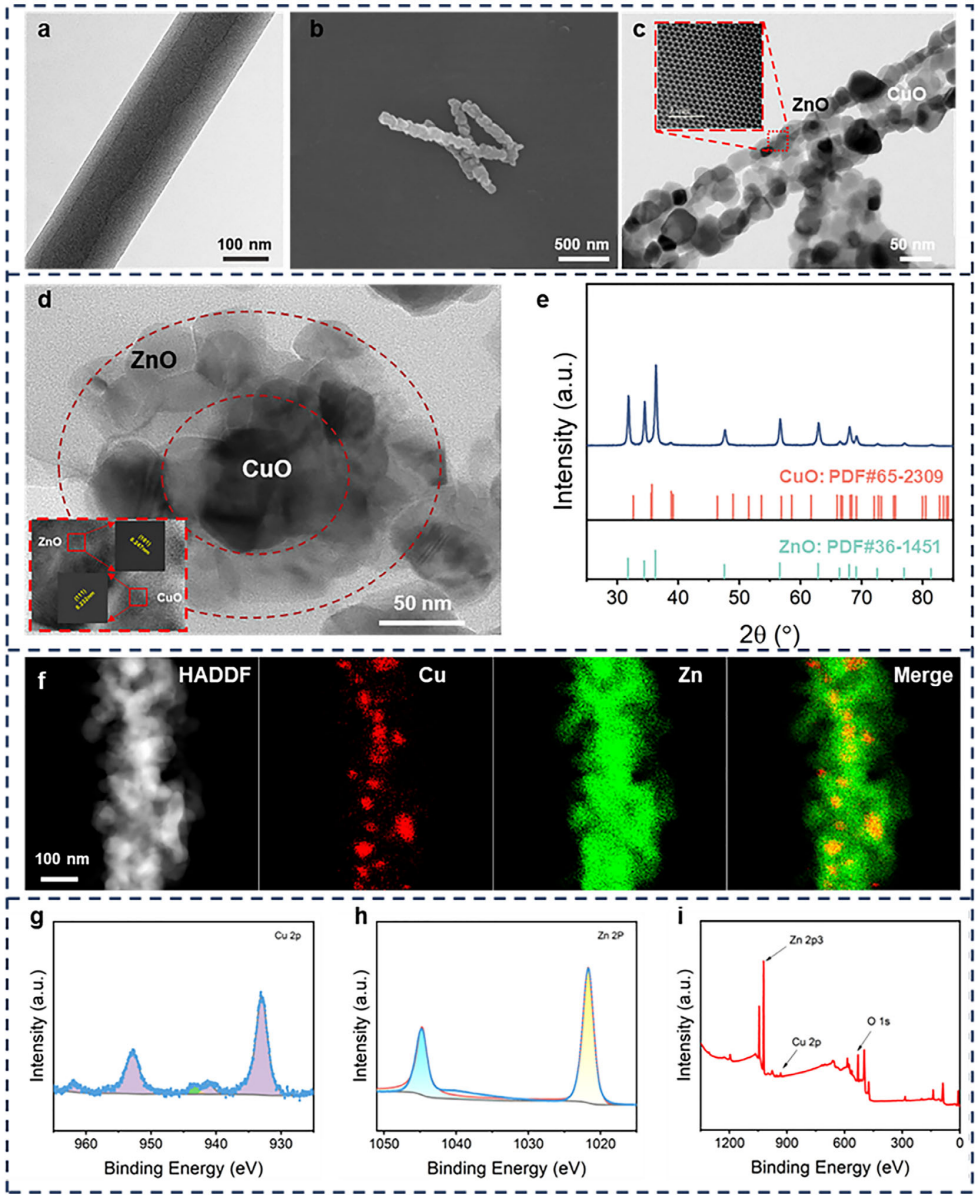
Characterization of the coaxial structure of ZnO@CuO nanofibers with inner CuO layer and outer ZnO layer. (a) TEM image of ZnO@CuO coaxial nanofiber precursor fibers. (b) SEM image of ZnO@CuO coaxial nanofibers after ultrasonic dispersion. HRTEM images of axial (c) and cross‐sectional (d) surfaces of ZnO@CuO coaxial nanofibers. (e) XRD pattern, (f) EDS mapping by AC‐TEM, and (g‐i) XPS patterns of ZnO@CuO coaxial nanofibers.

Coaxial nanofibers were prepared using a coaxial electrospinning system combined with high‐temperature calcination (Figure S1). ZnO@CuO nanofibers with different CuO ratio gradients in the inner layer were constructed, and the coaxial bonding morphology was observed using transmission electron microscopy ‌(TEM) (Figure S2). The optimal CuO content was 40 wt%. TEM was used to observe the precursor fibers of the ZnO@CuO coaxial nanofibers, and the core and shell layers are visible (Figure 2a). To characterize the coaxial structure of the ZnO@CuO coaxial nanofibers, ultrathin slices were used to obtain the cross sections. High‐resolution TEM (HRTEM) images showed that ZnO in the shell layer surrounded CuO in the core layer (Figure 2d). Obvious lattice stripes were observed at the junction of the core and shell with facet spacings of 0.247 and 0.232 nm, corresponding to (101) for ZnO and (111) for CuO, respectively [34]. The axial surface of the ZnO@CuO nanofibers was observed by TEM, and there was a clear demarcation between the core and the shell (Figure 2c). Scanning electron microscopy (SEM) was used to observe the morphology of the coaxial nanofibers before and after ultrasonic dispersion (Figure 2b; Figure S3). The fiber morphology of ZnO@CuO remains intact, indicating the stability of the coaxial structure. The atomic arrangement of the ZnO shell was observed using aberration‐corrected scanning transmission electron microscopy (AC‐STEM). X‐ray diffraction (XRD) was used to investigate the crystal and phase structure of ZnO@CuO coaxial nanofibers, and the result showed that peaks of the pattern at 2θ = 31.86°, 34.56°, 36.36° were faultlessly indexed to the (100), (002), and (101) planes of ZnO (JCPDS: 36–1451), respectively (Figure 2e). In addition, additional peaks at 2θ value of 35.54°, 38.82°, and 58.34° were corresponding to (002), (111), and (202) planes of monoclinic phase CuO (JCPDS: 65–2309). Moreover, characteristic peaks at approximately 440 and 547 cm‐1 were observed in the Fourier transform infrared (FTIR) spectra of the ZnO@CuO coaxial nanofibers, which also confirmed the presence of ZnO and CuO (Figure S4) [35, 36, 37, 38]. Subsequently, the elemental composition of the ZnO@CuO coaxial nanofibers was characterized by energy dispersive spectrometry (EDS) mapping, which revealed that Cu was located on the central axis of Zn, indicating the establishment of the ZnO@CuO coaxial structure (Figure 2f). X‐ray photoelectron spectroscopy (XPS) was performed to study the electronic and oxidation states of the elements of nanocomposites (Figure 2g–i), which confirmed the corresponding binding energies of 1021.24 eV (Zn 2p3), 932.2 eV (Cu 2p), and 531.03 eV (O 1s) [34].

### 2.2 Photocatalytic Performance of ZnO@CuO Nanofibers Enhanced by the Coaxial Structure

**FIGURE 3:**
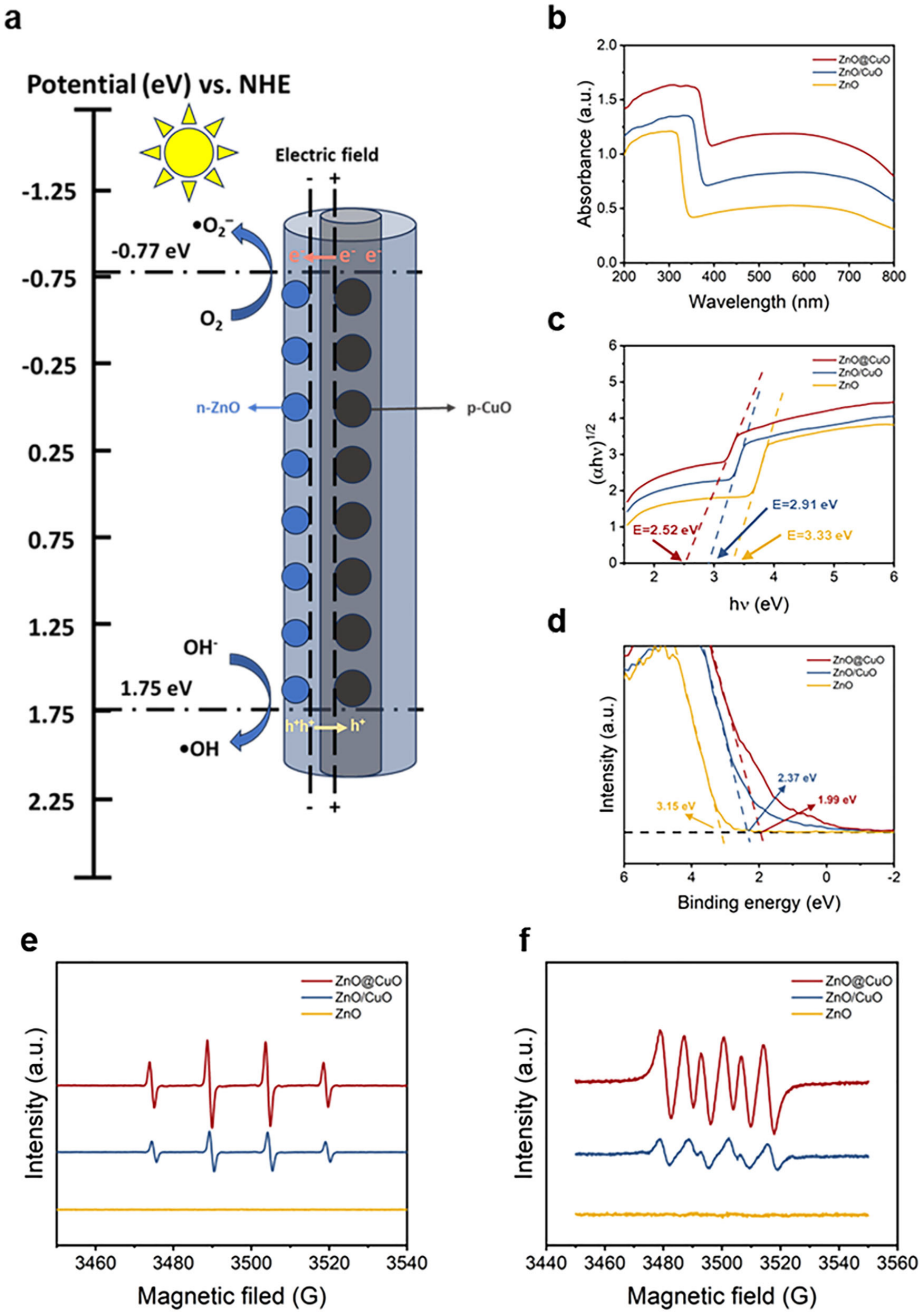
ZnO@CuO nanofibers enabled photocatalysis reactions under visible light, and the photocatalytic performance was enhanced by the coaxial structure. (a) Photocatalytic reaction schema of ZnO@CuO coaxial nanofiber heterojunction. (b) UV‐VIS spectra, (c) Tauc curve, (d) VB‐XPS patterns of ZnO@CuO coaxial nanofibers, ZnO/CuO nanofibers and ZnO nanofibers. EPR spectras of (e) DMPO−•OH and (f) DMPO−•O_2_^−^ adducts in the photocatalytic systems of ZnO@CuO coaxial nanofibers, ZnO/CuO nanofibers and ZnO nanofibers after 5 min irradiation of the light.

The most probable photocatalytic mode of the ZnO@CuO coaxial nanofibers is shown in Figure 3a. To investigate the effect of the coaxial structure on photocatalytic performance, we used ZnO/CuO nanofibers without a coaxial structure (ZnO/CuO) and ZnO nanofibers (ZnO) as control groups. The ultraviolet‐visible (UV‐VIS) spectra of the three groups are shown in Figure 3b to study the semiconductor energy band gaps; the ZnO@CuO coaxial nanofibers had the highest absorption peaks in the visible light absorption band from 400 to 700 nm [39]. The Tauc curve calculated from the UV‐VIS spectra showed that the ZnO@CuO coaxial nanofibers had the narrowest energy bandgap (2.52 eV, which allowed for photocatalytic reactions in the visible wavelength band (E_g_ < 3.2 eV) (Figure 3c) [40]. Owing to the incorporation of CuO, the energy band gap of the material system narrowed, which facilitated the photocatalytic reaction under visible light. From the XPS valence band (VB‐XPS) spectra of the ZnO@CuO coaxial nanofibers (Figure 3d), the valence band potential (E_VB_) was 1.99 eV. The EVB of ZnO@CuO coaxial nanofibers versus the NHE was calculated as 1.75 eV, and the conduction band potential (E_CB_) versus the NHE was further calculated as −0.77 eV, as shown in Figure 3a [41].

To further investigate the reactive substances produced in the photocatalytic reaction, electron paramagnetic resonance (EPR) spin‐capture experiments were performed on the ZnO@CuO coaxial nanofibers under visible‐light irradiation [42]. As shown in Figure 3e, the ·OH signal generated by ZnO@CuO coaxial nanofibers and ZnO/CuO nanofibers but not ZnO nanofibers was captured by 5,5‐dimethyl‐1‐pyrroline N‐oxide (DMPO) after visible light irradiation, and the ·OH signal of ZnO@CuO coaxial nanofibers was more intense. This might contribute to the accumulation of more active photogenerated holes on the valence band of ZnO@CuO coaxial nanofibers, and then promoted the oxidation reaction and increased the production of the ·OH radical (Figure 3a) [43]. Similarly, the ZnO@CuO coaxial nanofibers produced more intense signals in the experiments of ·O_2_^−^ capture (Figure 3f), due to more active electrons residing over the conduction band hastily facilitating the ·O_2_^−^ radical from dissolved oxygen (Figure 3a) [43]. These results indicate that the ZnO@CuO coaxial nanofibers could generate ROS under visible‐light photocatalysis, and the coaxial structure had a higher photocatalytic efficiency than the other two structures.

Given that the pH of the infectious wound microenvironment generally varies from 7.5 to 8.9 [44], a pH‐sensitive ROS generation test was conducted to mimic in vivo conditions. The EPR results showed that visible light‐regulated ZnO@CuO coaxial nanofibers maintained high photocatalytic activity in the pH range of 7.0 to 9.0 (Figure S5). This indicates that ROS generation in our material was stable under infectious wound conditions.

### 2.3 Photogenerated Carrier Dynamics of Heterojunctions Optimized by Coaxial Structure of ZnO@CuO Nanofibers

**FIGURE 4:**
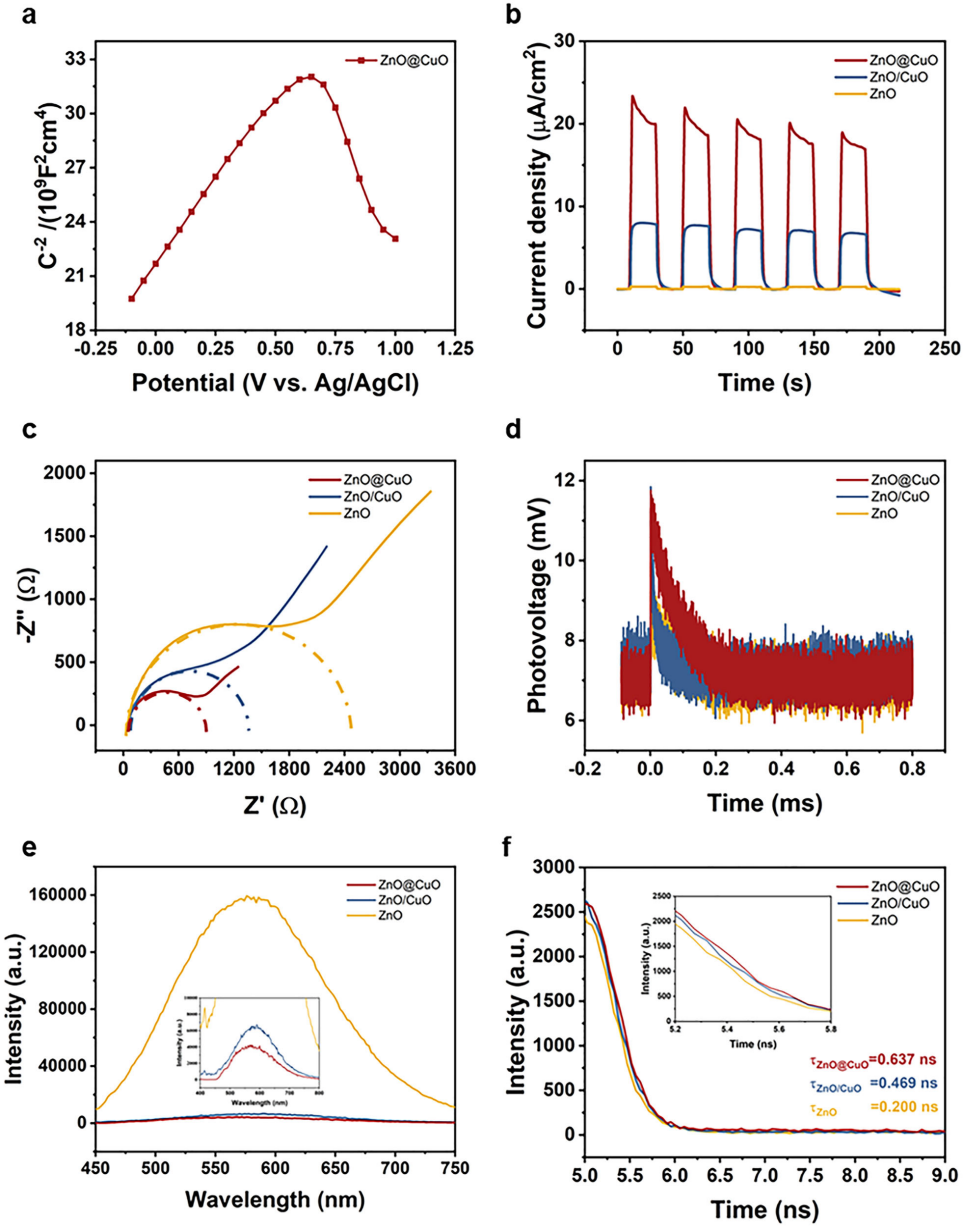
Photogenerated carrier dynamics were optimized by the coaxial structure of ZnO@CuO nanofibers. (a) Mott‐Schottky plot of ZnO@CuO coaxial nanofiber with heterojunctions. (b) Transient photocurrent responses, (c) EIS, (d) TPV, (e) PL spectra and (f) TRPL spectra of ZnO@CuO coaxial nanofibers, ZnO/CuO nanofibers, and ZnO nanofibers.

To confirm the formation of p‐n heterojunctions in the ZnO@CuO coaxial nanofibers, Mott–Schottky measurements were performed (Figure 4a). The results show that the positive slope of the Mott–Schottky curve of the ZnO@CuO coaxial nanofibers corresponds to the structure of the n‐type semiconductor ZnO, and the negative slope corresponds to the structure of the p‐type semiconductor CuO. The presence of both positive and negative slopes in the Mott‐Schottky plot is a typical characteristic of p‐n heterojunctions, indicating that p‐n heterojunctions were successfully constructed in the ZnO@CuO coaxial nanofibers [45].

To further explore the mechanism of enhanced photocatalysis by the ZnO@CuO coaxial nanofiber heterojunctions, the dynamics of the heterojunction‐photogenerated carriers were investigated using both photoelectrochemistry and spectroscopy. Figure 4b shows the transient photocurrent response for multiple on‐off cycles for the three groups. The ZnO@CuO coaxial nanofibers exhibited a significant photocurrent density compared to the pure ZnO and ZnO/CuO nanofibers, indicating a more efficient separation of photogenerated electron‐hole pairs and a faster transfer rate [43]. Similarly, the ZnO@CuO coaxial nanofibers exhibited the smallest interfacial transfer resistance in the electrochemical impedance test (Figure 4c), indicating that the photogenerated carriers encountered less transfer resistance during migration from the bulk to the surface [43]. This suggests that the construction of the coaxial structure could promote the efficient transfer of photogenerated carriers through the built‐in electric field of the heterojunction, which would give the photogenerated carriers a greater possibility of participating in the photocatalytic reaction [32]. To investigate the transport behavior of the photogenerated carriers further, we performed a transient photovoltage (TPV) test (Figure 4d). The results showed that the photovoltage of the ZnO@CuO coaxial nanofiber with heterojunctions decreased slowly after the peak of the photovoltage response, which corresponded to the slow compounding process of the electrons leaving the conducting substrate [46, 47]. This further illustrates that the ZnO@CuO coaxial nanofibers have a longer electron presence during photocatalysis than the ZnO/CuO nanofibers, which also implies better electron‐hole pair separation.

Subsequently, the dynamics of photogenerated carriers in the heterojunction was investigated from a spectroscopic point of view by photoluminescence spectroscopy and fluorescence lifetime spectroscopy [48, 49]. The ZnO@CuO coaxial nanofiber heterojunction exhibited the lowest peak in the photoluminescence spectra, indicating the lowest electron‐hole pair complexation and the highest separation efficiency (Figure 4e). Finally, time‐resolved photoluminescence spectroscopy was used to calculate the fluorescence lifetimes of three nanofibers. The ZnO@CuO coaxial nanofiber heterojunction exhibits the longest fluorescence lifetime (0.637 ns) (Figure 4f). Consistent with the results of photoluminescence spectroscopy, the longer fluorescence lifetimes indicated that the electrons were present for a longer time, which in turn suggested that the coaxial structure enhanced the efficiency of the separation of electron‐hole pairs [49]. The above results illustrate the optimization of the photocatalytic performance of the coaxial structure by improving the photogenerated carrier dynamics and enhancing the separation efficiency of electron‐hole pairs.

### 2.4 Biocompatibility and Antimicrobial Properties of ZnO@CuO Coaxial Nanofibers Under Visible Light In Vitro

The CCK‐8 test was used to determine the biologically safe concentration of ZnO@CuO coaxial nanofibers with heterojunctions for in vitro antimicrobial assays (Figure S6). ZnO@CuO coaxial nanofibers at a biocompatible concentration of 20 µg/mL were used for subsequent biological experiments. ZnO, a commonly used clinical antibacterial agent, was used to compare the antibacterial properties of ZnO@CuO coaxial nanofibers. An LDH assay was conducted to evaluate the biocompatibility of the coaxial ZnO@CuO nanofibers. The result showed that all groups exhibited good biosafety, and the number of viable cells in the coaxial ZnO@CuO nanofiber group was higher than that in the ZnO/CuO nanofiber group (Figure S7), indicating that the coaxial structure provided superior biosafety.

**FIGURE 5:**
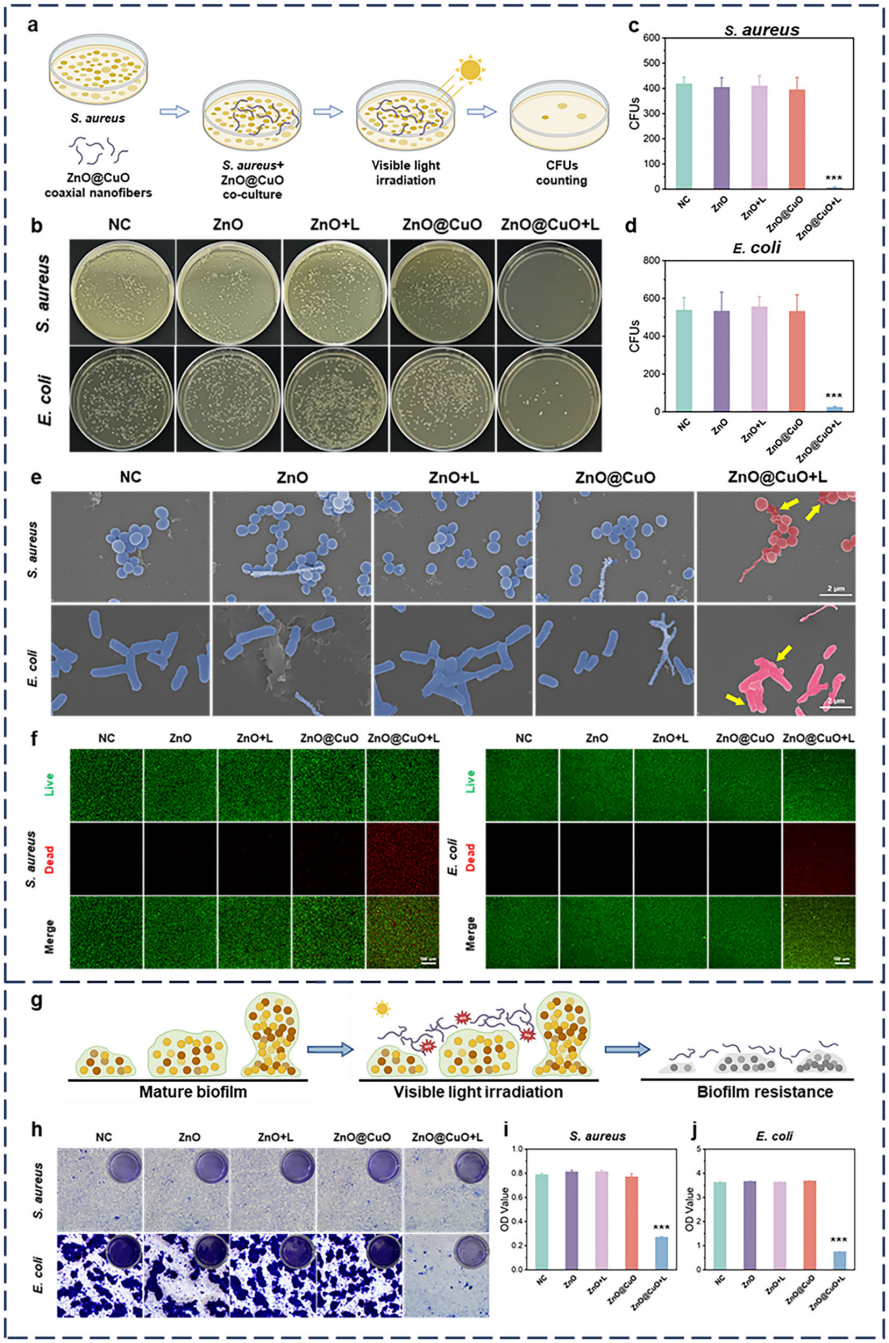
ZnO@CuO coaxial nanofibers exhibited excellent antimicrobial properties under visible light in vitro. (a) Schematic of in vitro antimicrobial activity of coaxial nanofibers. Created with BioRender.com. (b) Plate‐coated images of the corresponding methicillin‐resistant *S. aureus* and *E. coli* after incubation in different groups. Quantitative analysis of methicillin‐resistant *S. aureus* (c) and *E. coli* (d) colonies in (b). (n = 3 biologically independent samples; mean ± SD; ***p < 0.001, one‐way ANOVA with Tukey's post hoc test). (e) SEM images and (f) Fluorescence images of live/dead staining of methicillin‐resistant *S. aureus* and *E. coli* morphologies after incubation in different groups. (g) Schematic of in vitro anti‐biofilm of coaxial nanofibers. Created with BioRender.com. (h) Violet staining images and biofilm biomass (i‐j) of methicillin‐resistant *S. aureus* and *E. coli* biofilm after different treatments. (n = 5 biologically independent samples; mean ± SD; ***p < 0.001, one‐way ANOVA with Tukey's post hoc test).

To explore the antibacterial activity of ZnO@CuO coaxial nanofibers under visible light, colony formation experiments and counting of colony‐forming units (CFUs) were performed on methicillin‐resistant *Staphylococcus aureus* (*S. aureus*) and *Escherichia coli* (*E. coli*) (Figure 5a). The results showed that the number of colonies formed by methicillin‐resistant *S. aureus* and *E. coli* was significantly reduced in the ZnO@CuO + L group (Figure 5b–d), whereas this effect was inhibited by the addition of N‐acetylcysteine (NAC), an ROS scavenger (Figure S8). This implies that the ZnO@CuO coaxial nanofibers can exert a strong antimicrobial effect via a photocatalytic reaction under visible light, and that this antibacterial effect is ROS‐dependent. Live/dead bacterial staining showed that the red fluorescence of dead methicillin‐resistant *S. aureus* and *E. coli* significantly increased in the ZnO@CuO + L group, which was consistent with the CFUs results (Figure 5f). SEM images showed that most methicillin‐resistant *S. aureus* and *E. coli* lost their normal cell membrane structure and began to rupture in the ZnO@CuO + L group, whereas the membrane integrity of the bacteria in the other groups was less affected. Interestingly, the ZnO@CuO coaxial nanofibers remained morphologically and structurally intact after interacting with methicillin‐resistant *S. aureus* and *E. coli* (Figure 5e), indicating good stability of the coaxial nanofibers.

To further investigate the destruction of bacterial biofilms by ZnO@CuO coaxial nanofibers under visible light, the biofilm biomass was evaluated by crystal violet staining (Figure 5g). A lighter purple color and lower biofilm biomass were observed in the ZnO@CuO + L group (Figure 5h). The OD statistics in Figure 5i,j quantitatively illustrate a significant reduction in the biofilm biomass of methicillin‐resistant *S. aureus* and *E. coli* in the ZnO@CuO + L group. Overall, the above results demonstrate that ZnO@CuO can produce better antibiofilm activity than ZnO in vitro under visible light.

### 2.5 Infected Skin Wound Healing Promoted by ZnO@CuO Coaxial Nanofibers Under Visible Light

**FIGURE 6:**
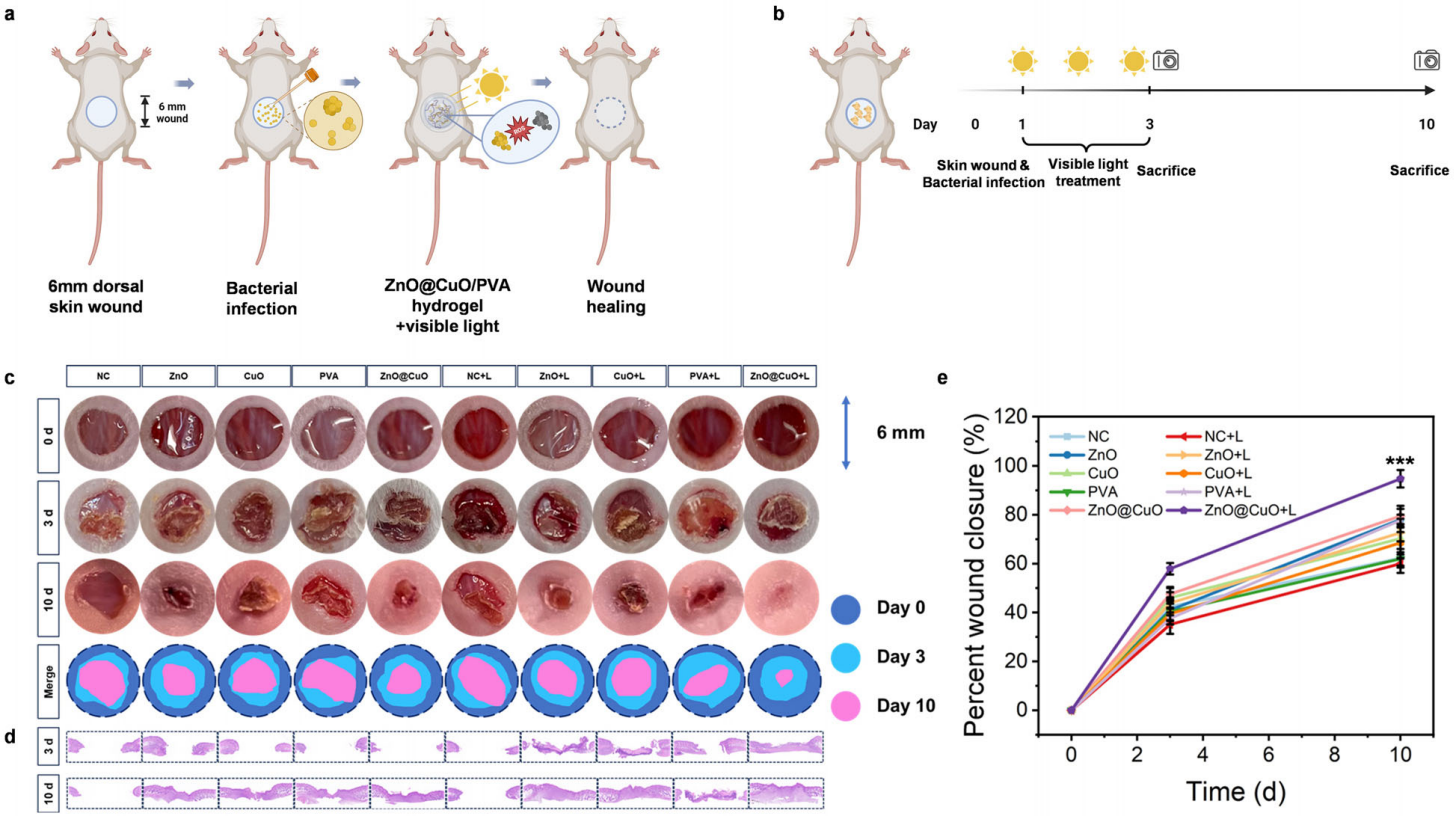
ZnO@CuO coaxial nanofibers enhanced infected wound healing in vivo. (a) Establishment of mouse skin defect model and (b) the following treatment schedule. Created with BioRender.com. (c) Photos of methicillin‐resistant *S. aureus* ‐infected Balb/c mice after different treatments and the scheme of wound healing process. (d) H&E staining results of wound healing process. (e) Wound closure curve of different treatments. (n = 3 mice for per group; mean ± SD; ***p < 0.001, one‐way ANOVA with Tukey's post hoc test).

To investigate the in vivo antibacterial activity of the ZnO@CuO coaxial nanofibers via photocatalysis under visible light, we established a 6‐mm dorsal skin defect model in BALB/c mice infected with methicillin‐resistant *S. aureus* and evaluated the wound‐healing effects (Figure 6a). Nanofibers in the three groups were loaded into a PVA hydrogel, a common biocompatible material, to prepare dressings to cover the wound surface, and visible light was applied (Figure 6b). Representative images of infected wounds from different groups (Figure 6c) show that bacterial infection delayed wound healing, whereas the ZnO@CuO + L group had the fastest wound healing effect. The quantitative statistics of wound closure are shown in Figure 6e, where the ZnO@CuO + L group achieved nearly 60% wound closure on day 3, and the wound was nearly completely healed on day 10, implying that the ZnO@CuO + L group significantly increased new tissue formation. H&E staining of skin tissue sections in the ZnO@CuO + L group showed the best performance in terms of the integrity and thickness of new skin tissue, indicating that the ZnO@CuO coaxial nanofibers under visible light can quickly eliminate bacteria, control infection in the early stage, and promote wound healing (Figure 6d).

**FIGURE 7:**
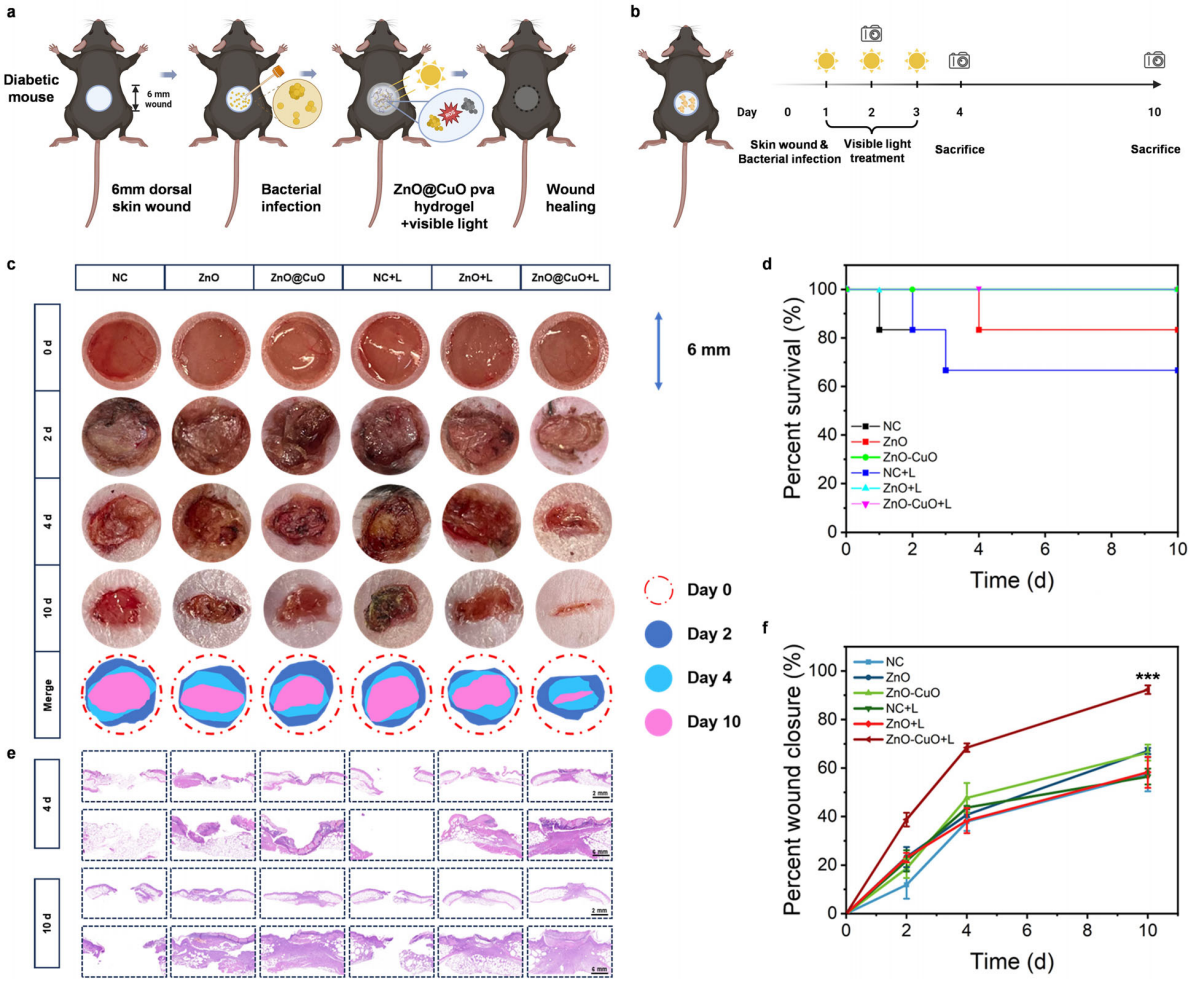
ZnO@CuO coaxial nanofibers enhanced infected diabetic wound healing in vivo. (a) Establishment of diabetic mouse skin defect model and (b) the following treatment schedule. Created with BioRender.com. (c) Photos of methicillin‐resistant *S. aureus* ‐infected db/db mice after different treatments and the scheme of wound healing process. (d) Survival curve of different treatments. (e) H&E staining results of wound healing process. (f) Wound closure curve of different treatments. (n = 3 mice for per group; mean ± SD; ***p < 0.001, one‐way ANOVA with Tukey's post hoc test).

To further validate the bactericidal and anti‐infective effects of the ZnO@CuO coaxial nanofibers in a diabetic environment, we constructed methicillin‐resistant *S. aureus*‐infected skin defect models in diabetic db/db mice (Figure 7a,b). As shown in the survival curve in Figure 7d, deaths in the NC and ZnO groups at the early stage of the experiment proved that infected skin defects with diabetes comorbidities may seriously endanger life [50]. Consistent with the healing trend in the normal mouse model, skin healing images and wound healing curves showed that the healing rate of the ZnO@CuO + L group was 38.8% on day 2 and 92.3% on day 10, which was significantly higher than that of the other groups (Figure 7c,f). This was because the visible light‐activated ZnO@CuO coaxial nanofibers achieved time‐controlled ROS release, generating localized ROS storms to eliminate bacteria, thereby reducing endogenous ROS levels and accelerating wound healing in the early stages. In vivo fluorescent imaging of diabetic wounds directly assessed ROS dynamics at the wound site (Figure S9). The results showed that coaxial ZnO@CuO nanofibers under visible light exhibited the highest ROS signals at the wound site from day 1 to day 3, indicating that visible light activated the coaxial ZnO@CuO nanofibers to generate an ROS storm and thus enhance antibacterial activity. In the late healing stage (days 7–10), ROS levels in the ZnO@CuO + visible light group were markedly lower than those in the other groups, suggesting that early infection control provides a physiological microenvironment for wound healing, as reflected by the decrease in endogenous ROS. The H&E staining results showed that the ZnO@CuO + L group had the best new skin regeneration and tissue thickness in the diabetic mice (Figure 7e). Given that excessive ROS accumulation may exacerbate oxidative stress in infectious diabetic wounds, the expression of 8‐hydroxy‐2’‐deoxyguanosine (8‐OHdG) (oxidative stress marker [51]) in wound regions at 4 days was tested (Figure S10). The lowest 8‐OHdG level was observed in the ZnO@CuO + L group among all the groups, indicating that the timely elimination of infection relieved oxidative stress at the initial stage of diabetic wound healing. This further demonstrated that the generation of ROS by the ZnO@CuO coaxial nanofibers under visible light was temporally controllable, and excessive ROS did not accumulate at the wound site. Moreover, the immunohistochemical images for TNF‐α (inflammation marker [52]) at 4 days showed the lowest expression levels in the ZnO@CuO + L group, indicating that inflammation was also relieved as the infection was cleared. These results demonstrate the excellent antibacterial and wound‐healing properties of the ZnO@CuO coaxial nanofibers under visible light in the high‐glucose microenvironment of diabetic comorbidities.

## 3 Conclusion

In this paper, we report a novel ZnO@CuO nanofiber material with a stable coaxial structure. The p‐n heterojunctions built inside the nanofibers enabled ZnO@CuO to perform photocatalytic reactions under visible light, making ROS generation more convenient and biocompatible. Moreover, the coaxial structure improves the assembly efficiency of the heterojunctions. This leads to improved electron‐hole separation, optimized photogenerated carrier dynamics, and enhanced photocatalytic performance, ultimately enabling more controllable and efficient ROS generation. The ZnO@CuO nanofibers responsive to visible light exhibited excellent antimicrobial properties in vitro. By establishing a methicillin‐resistant *S. aureus*‐infected mouse skin defect model, we demonstrated that ZnO@CuO coaxial nanofibers could rapidly inhibit early stage wound infection and significantly accelerate defect healing through visible‐light photocatalysis, even under diabetic conditions. This antimicrobial wound dressing offers a convenient and biologically safe therapeutic strategy for the clinical treatment of non‐healing infected wounds.

## 4 Materials and Methods

### 4.1 Fabrication of ZnO@CuO Coaxial Nanofibers

1.5 g of zinc nitrate and 0.4 g of copper nitrate were dissolved in 10 mL of N,N‐dimethylformamide (DMF). 1.6 g of polyvinylpyrrolidone (PVP) was added to the zinc nitrate solution, and 0.5 g of PVP was added to the copper nitrate solution and stirred at room temperature for 6 h until complete dissolution to form the outer and inner precursor solutions for coaxial electrospinning. The precursor solutions were subjected to coaxial electrospinning at a voltage of 15 kV, an electrode spacing of 12 cm, a flow rate of 1 mL/h for the outer layer and 0.2 mL/h for the inner layer for a period of 8 h. The electrospun fibrous membrane was collected and then placed in a muffle furnace at 500°C for 4 h to obtain ZnO@CuO coaxial nanofibers. ZnO/CuO nanofibers were prepared by single‐axial electrospinning after mixing the above mentioned inner and outer precursor solutions, and pure ZnO nanofibers were prepared by single‐axial electrospinning with only the outer precursor solution.

### 4.2 Characterization of ZnO@CuO Coaxial Nanofibers

The morphology of the axial surface of the coaxial electrospinning precursor nanofibers, the axial surface, and cross‐section of the ZnO@CuO coaxial nanofibers were observed using a transmission electron microscope (TEM) (FEI Tencnai G2 F30, USA). The morphology of the ZnO@CuO coaxial nanofibers after being dispersed by ultrasound was analyzed using a field emission scanning electron microscope (FE‐SEM, S‐4800, HITACHI, Japan). The atomic arrangement of ZnO in the outer ZnO layer of the ZnO@CuO coaxial nanofibers was analyzed using aberration‐corrected scanning transmission electron microscopy (AC‐STEM, JEM‐ARM300F, Japan) equipped with an X‐ray energy dispersive spectroscopy (EDS) analyzer to determine the elemental composition and coaxial structure. The crystal and phase structure of the ZnO@CuO coaxial nanofibers was characterised by X‐ray diffraction spectroscopy (XRD, Rigaku D/max 2500VB2t/PC, Japan) using a 1.54 Å Cu Kα source. The chemical bonding of the coaxial nanofibers was analyzed using Fourier transform infrared spectroscopy (FTIR, SP400, Norwalk, CT, USA). The elemental oxidation states and the valence band of ZnO@CuO coaxial nanofibers were investigated using X‐ray photoelectron spectroscopy (XPS, Thermo Scientific K‐Alpha, USA). A UV‐VIS spectrophotometer (Shimadzu 3600) was used to record the reflectance spectra of all the nanofibers. Tauc curves were plotted after calculations based on UV–vis spectra. The equation for the conversion of E_VB_ to normal hydrogen electrode (NHE) potential was as follows [41, 53]:

$$E_{VB,\;NHE}=\varphi +E_{VB,\;XPS\;}-\;4.44$$

$$E_{VB}=E_{CB}+E_{g}$$

where the φ was the work function of the XPS instrument (φ = 4.2 eV), and E_g_ was derived from the value obtained in the Tauc curve.

### 4.3 Electronic Paramagnetic Resonance (EPR) Measurements

DMPO spin‐capture EPR spectra were recorded to directly detect hydroxyl radical adducts (DMPO‐·OH and superoxide radical adducts (DMPO‐·O_2_^−^) during degradation. Specifically, 5 mg of nanofibers and 50 µL of DMPO were dispersed in 1.5 mL of deionised water to detect DMPO‐·OH. On the other hand, 5 mg of nanofibers and 50 µL of DMPO were dispersed in 1.5 mL of CH_3_OH to detect the DMPO‐·O_2_^−^.

### 4.4 Photoelectrochemical Measurements

Mott‐Schottky measurements, electrochemical impedance spectroscopy (EIS) and transient photocurrent (I‐t) measurements were carried out using a CHI 660 electrochemical workstation (CHI Instruments, Shanghai), whose conventional three‐electrode system consists of a modified FTO electrode, an Ag/AgCl/saturated‐KCl electrode, and a Pt electrode (1 × 1 cm^2^), which serve as the working, reference, and auxiliary electrodes, respectively. The electrochemical setup consisted of a custom‐made square‐shaped electrochemical device. The electrochemical setup was made of a customised square quartz vessel (size 5 × 4 × 8 cm^3^). To pre‐treat the FTO electrodes, the FTOs were sonicated using toluene (15 min), acetone (20 min), ethanol (20 min), and deionised water (30 min). The light source for the tests was a 300 W Xe lamp (λ ≥ 420 nm). Typically, 10 mg of nanofibers was dissolved in 2 mL ethanol to form the emulsions. Mott–Schottky measurements are recorded under 25 mV applied bias over a frequency of 500 Hz under dark. EIS tests were performed at 10^4^‐10^2^ Hz in Fe(CN)_6_^3−/4−^ solution (pH = 8.05) containing KCl solution (1 mmol L^−1^) with different open‐circuit voltages applied at different catalyst/FTO electrodes. Transient I‐t was tested in 0.2 mol L^−1^ Na_2_SO_4_ electrolyte (pH = 7.86) with an applied potential of 5 mV (compared to Ag/AgCl). The transient photovoltage (TPV) test was measured on a surface photovoltage spectrometer. PL spectra and fluorescence lifetime spectra were obtained on a steadily transient fluorescence spectrometer (FLS1000/FS5, Edinburgh, UK).

### 4.5 Biocompatibility Tests

To assess biocompatibility, a Cell Counting Kit‐8 (CCK‐8) assay (DOJINDO, Kumamoto, Japan) was performed. L929 (NCTC clone 929, Procell) cells (1 × 10⁴ per well) were randomly inoculated into 96‐well plates and co‐cultured with different concentrations of ZnO@CuO coaxial nanofibers by incubation at 37°C for 24 h. After 1, 2, and 3 days of incubation, cells were treated with 10% CCK‐8 reagent for 2 h at 37°C. Absorbance was measured at 450 nm using a microplate reader (Biotek Synergy Neo2, Winooski, Vermont). To further evaluate biocompatibility, an LDH cytotoxicity assay (Beyotime, Shanghai, China) was performed. L929 (NCTC clone 929, Procell) cells (1 × 10⁴ per well) were randomly seeded into 96‐well plates and co‐cultured with different groups of nanofibers at 37°C for 24 h. LDH release was measured according to the manufacturer's instructions, and absorbance was recorded at 490 nm using a microplate reader (Biotek Synergy Neo2, Winooski, Vermont).

### 4.6 In Vitro Antimicrobial Assays

The in vitro antimicrobial assays were performed using the Gram‐positive bacterium methicillin‐resistant *S. aureus* (MRSA) (ATCC 43300, Thermo Fisher Scientific) and the Gram‐negative bacterium *E.coli* (ATCC 25922, Thermo Fisher Scientific). 1 mL of bacteria (10^6^ CFU mL^−1^ for MRSA and 10^6^ CFU mL^−1^ for *E. coli*) were randomly treated with different groups (NC, ZnO, ZnO + L, ZnO@CuO, and ZnO@CuO + L) for 24 h at 37°C, respectively. The in vitro antimicrobial activities of the groups against MRSA and *E. coli* were evaluated by colony counting, live/dead bacterial staining (Thermo Fisher, USA), and scanning electron microscopy (SEM). In the anti‐biofilm assay, mature biofilms treated in the different groups described above were stained with crystal violet (1%, w/v) for 15 min. Then, the stained biofilms were rinsed slightly with pure water three times to remove excess crystal violet. The crystalline violet on the biofilms was dissolved by incubation with 33% acetic acid at 37°C for 30 min. Finally, the biomass of the biofilm was assessed by measuring the absorbance at 590 nm using a microplate reader.

### 4.7 Infected Wound Healing Accelerated by ZnO@CuO/PVA Hydrogel

All animal surgical procedures were approved by the Institutional Animal Care and Use Committee of Peking University Health Science Center (No. DLASBD0443). Balb/c mice (male, 6 weeks) were purchased from Beijing Vital River Laboratory Animal Technology Co., Ltd. The mice in the animal experiments were randomly assigned to each group for different treatments. For surgical excision of the wound, mice were first anesthetized by intraperitoneal injection of sodium pentobarbital (0.1 mL/20 g) and shaved dorsally. A full‐thickness skin wound with a diameter of 6 mm was made on the back of each mouse. Then, 10 µL of methicillin‐resistant *S. aureus* (10^6^ CFUs/mL) bacterial solution was inoculated onto this wound. Different nanofibers loaded into 40% wt of PVA hydrogel were applied to the wound. The mice wounds were divided into ten groups (n = 3) with light treatment marked as “+L”: negative control group (NC), NC + L, PVA hydrogel group (PVA), PVA + L, ZnO nanofibers + PVA hydrogel group (ZnO), ZnO +L, CuO nanofibers +PVA hydrogel group (CuO), CuO + L, ZnO@CuO coaxial nanofibers +PVA hydrogel group (ZnO@CuO), and ZnO@CuO + L. 30 min, 300 W Xe lamp simulating sunlight was given to the +L groups on days 1, 2, and 3 of the application of hydrogel. Samples were taken on days 3 and 10 for histopathological sections and H&E staining.

Diabetic mice db/db (male, 6 weeks old) were purchased from cyagen bio (Suzhou, China). The infected wound model was consistent with Balb/c mice. The mice in the animal experiments were randomly assigned to each group for different treatments. Mouse wounds were divided into 6 groups (n = 3) with light treatment labelled ‘+L’: negative control (NC), NC+L, ZnO nanofibers + PVA hydrogel (ZnO), ZnO +L, ZnO@CuO coaxial nanofibers + PVA hydrogel (ZnO@ CuO), and ZnO@CuO + L. The +L group was given 30 min, 300 W Xe lamp to simulate sunlight illumination on days 1, 2, and 3 of hydrogel application. Samples were taken on days 4 and 10 for histopathological sections and H&E staining. To assess in vivo ROS generation, the wound sites were stained with a DCFH‐DA fluorescent probe (Solarbio, China) on days 1, 2, 3, 7, and 10 according to the manufacturer's instructions. Fluorescence signals were captured using an IVIS Spectrum Living Imaging System (PerkinElmer, USA), and the images were analyzed using Living Imaging software. Immunohistochemical staining was conducted at the skin wound sites of db/db mice on day 4 to evaluate oxidative stress and inflammation. Tissue sections were stained with anti‐8‐OHdG antibody (Abcam, ab62623) and anti‐TNF‐α antibody (Servicebio, GB115702‐50) following standard protocols.

### 4.8 Statistical Analysis

All statistical analyses were evaluated by the Origin (2022) software. Results were presented as mean ± standard deviation (SD, represented as error bars). Statistical analyses were performed using one‐way ANOVA with Tukey's post hoc test. Statistical significance was indicated by three asterisks (*p* < 0.001).

## Conflicts of Interest

The authors declare no conflicts of interest.

## Supporting information




**Supporting File**: advs73413‐sup‐0001‐SuppMat.docx.

## Data Availability

The data that support the findings of this study are available from the corresponding author upon reasonable request.
