# Supplementary material for: Visible Light‐Activated ZnO@CuO Coaxial Nanofibers Enhance Infected Skin Wound Healing Through Construction of p‐n Heterojunctions
Source: Adv Sci (Weinh). 2026 Jan 8;13(16):e12700. doi: 10.1002/advs.202512700 (PMC13042498; doi:10.1002/advs.202512700)
Supplement: Supplementary file 1 — Supporting File: advs73413‐sup‐0001‐SuppMat.docx. [file ADVS-13-e12700-s001.docx]

**Supplementary Information**

**Visible Light-Activated ZnO@CuO Coaxial Nanofibers Enhance Infected Skin Wound Healing through Construction of p-n Heterojunctions**

*Pengrui Dang^1, 2#^, Han Zhao^3#^, Chenguang Zhang^4#^, Jiechen Wang^5^, Fangyu Zhu^1^, Qinqiu Zhong^2^, Wenyi Zeng^4^, Xinyuan Wang^5^, Yumin Chen^1^, Xu Yan^2*^, Xuliang Deng^1*^, Wenwen Liu^1*^*

1. Department of Geriatric Dentistry, Peking University School and Hospital of Stomatology & National Center for Stomatology & National Clinical Research Center for Oral Diseases & National Engineering Research Center of Oral Biomaterials and Digital Medical Devices & NMPA Key Laboratory for Dental Materials, No.22, Zhongguancun South Avenue, Haidian District, Beijing, 100081, China.

2. The VIP Department, School and Hospital of Stomatology, China Medical University, Liaoning Provincial Key Laboratory of Oral Diseases, No.106, North Second Road, Heping District, Shenyang, 110002, China.

3. Department of General Dentistry, Peking University School and Hospital of Stomatology & National Center for Stomatology & National Clinical Research Center for Oral Diseases & National Engineering Research Center of Oral Biomaterials and Digital Medical Devices & NMPA Key Laboratory for Dental Materials, No.22, Zhongguancun South Avenue, Haidian District, Beijing, 100081, China.

4. Hospital of Stomatology, Guanghua School of Stomatology, Sun Yat-sen University, Guangdong Provincial Key Laboratory of Stomatology, Guangzhou, 510275, China.

5. Department of Stomatology, Union Hospital, Tongji Medical College, Huazhong University of Science and Technology, Wuhan, 430022, China.

*Corresponding authors:

Wenwen Liu, wenwendentist@gmail.com;

Xuliang Deng, kqdengxuliang@bjmu.edu.cn;

Xu Yan, xyan@cmu.edu.cn.

#These authors contributed equally to this work.

**Table S1 Wound contraction efficiency and ROS strategies for referenced materials**

| Material name | ROS strategy | Light type | PTT synergism | Wound contraction efficiency | References |
| --- | --- | --- | --- | --- | --- |
| PB@PDA@Ag | Generating | NIR | Yes | 60% | [1] |
| BPQDs@NH | Generating | NIR | Yes | 80% | [2] |
| Au NCs@PCN | Generating | NIR | Yes | 60% | [3] |
| TMB/Fe^2+^/PF127/GOx | Generating | NIR | Yes | 87.70% | [4] |
| MXene@PDA | Scavenging | NIR | Yes | 98.80% | [5] |
| PDPC2 | Scavenging | NIR | Yes | 95.90% | [6] |
| Nb_2_C@Gel | Scavenging | NIR | Yes | 55% | [7] |
| Gel-HAB | Scavenging | NIR | Yes | 85% | [8] |
| MnO_2_@PDA-BGs/Gel | Scavenging | NIR | Yes | 82.32% | [9] |
| MoS_2_-CeO_2_ | Scavenging | NIR | Yes | 90% | [10] |
| OGF | Scavenging | NIR | Yes | 70% | [11] |
| PAG-CuS | Scavenging | NIR | Yes | 60% | [12] |
| DCPM | Scavenging | NIR | Yes | 80% | [13] |
| CDP-PB | Scavenging | NIR | Yes | 80% | [14] |
| 4OI–BP@Gel | Generating  then scavenging | NIR | Yes | 82.97% | [15] |
| Au/AgNDs@Gel | Not involved | NIR | Yes | 30% | [16] |
| PDA/Cur NFs | Not involved | NIR | Yes | 81.80% | [17] |
| PVA-CS-HTCC-PANI-GSNO | Not involved | NIR | Yes | 90% | [18] |
| MGACS | Not involved | NIR | Yes | 70% | [19] |
| MCC/CS NPs | Not involved | NIR | Yes | 84% | [20] |
| F-ZnO@Ag NPs | Not involved | NIR | Yes | 80% | [21] |
| HP/BP-Zn^2+^@TA | Not involved | NIR | Yes | 70% | [22] |
| Gel/PL@Fe^III^TA | Not involved | NIR | Yes | 70% | [23] |
| M-HMP MNs | Not involved | NIR | Yes | 84% | [24] |
| COA-T3 | Generating | Visible light | No | 85.20% | [25] |
| Ce6@EnHOF-101 | Generating | Visible light | No | 90% | [26] |
| TSeL | Generating  then scavenging | Visible light | No | 86.67% | [27] |
| PTMH | Not involved | Visible light | No | 62.50% | [28] |
| PMH | Not involved | Visible light | No | 85% | [29] |

**Table S1. Studies evaluating the efficiency of wound contraction using a diabetic mouse model of infected skin defects were included.** The results of all included biomaterials as well as ZnO@CuO/PVA under this model were shown in Figure 1a.


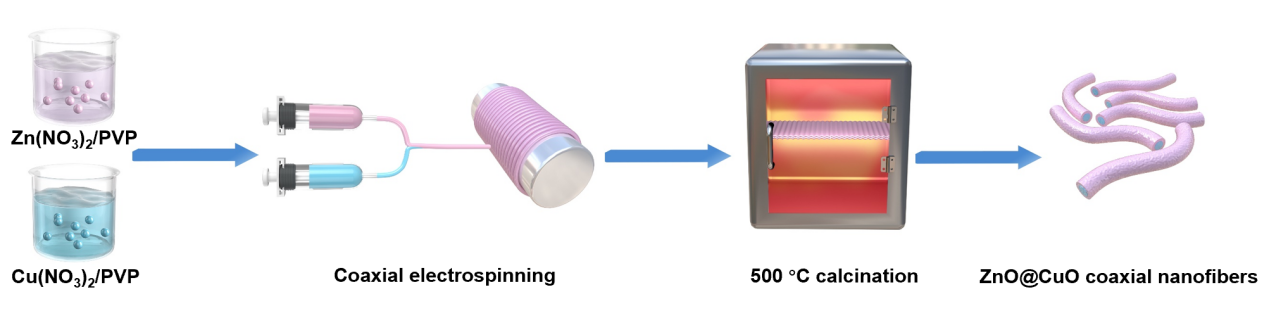


**Figure S1. Schematic illustration of the fabrication of ZnO@CuO coaxial nanofibers.**


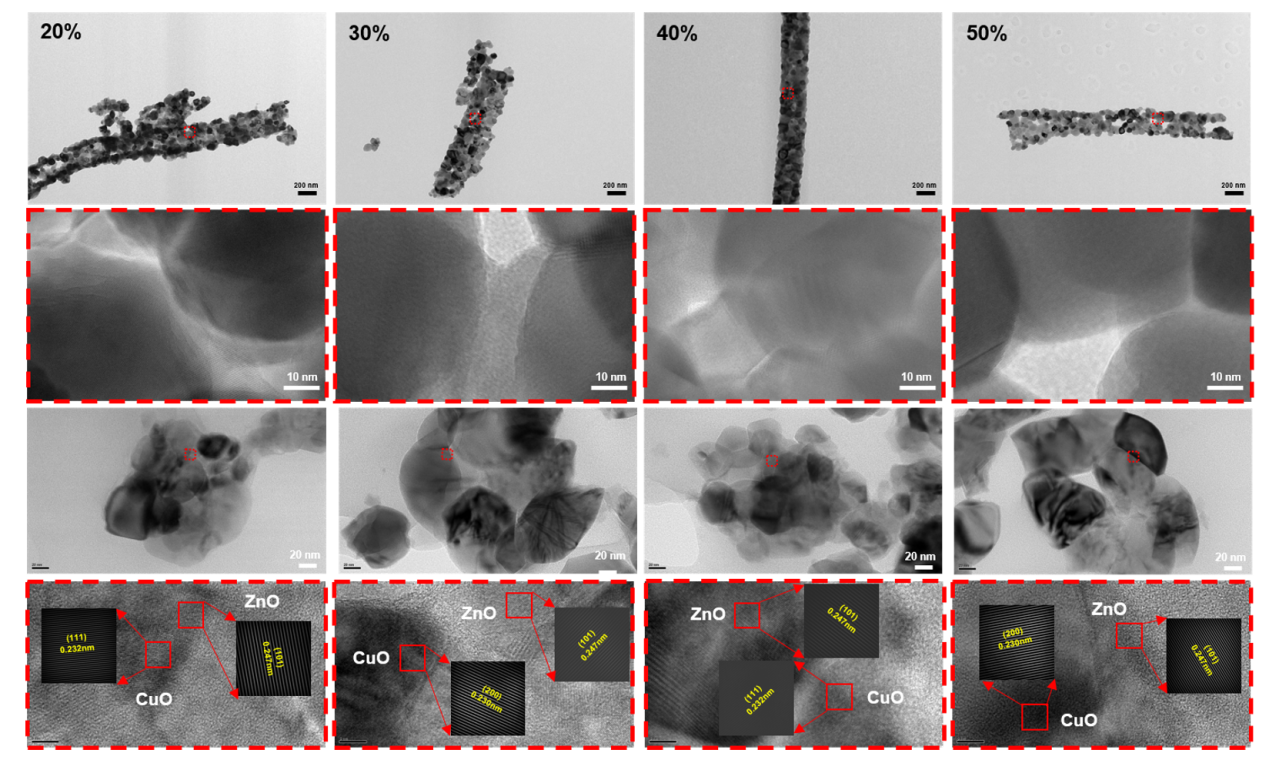


**Figure S2. TEM images of ZnO@CuO nanofibers with different CuO ration gradients.**

**
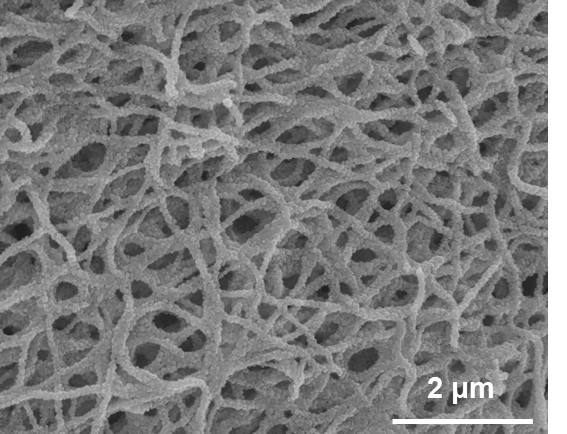
**

**Figure S3. SEM image of ZnO@CuO coaxial nanofibers.**

**
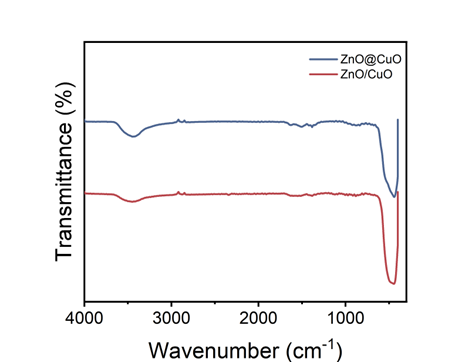
**

**Figure S4. FTIR spectra of ZnO@CuO coaxial nanofibers and ZnO/CuO nanofibers.**

**Figure S5. EPR spectras of (a) DMPO−•OH and (b) DMPO−•O_2_^–^ adducts in the photocatalytic system of ZnO@CuO coaxial nanofibers at pH = 7, 8, and 9.
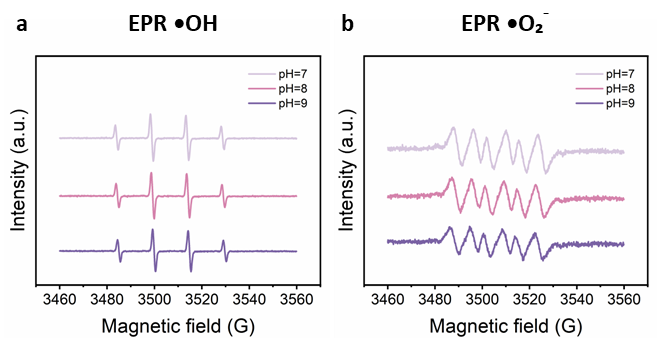
**

**
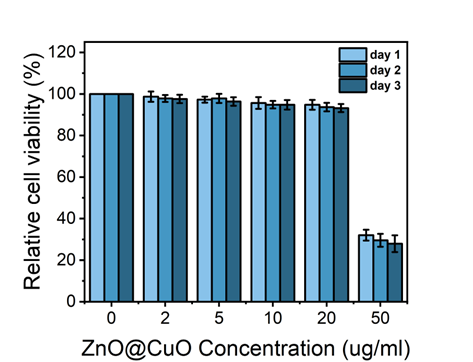
**

**Figure S6. Relative cell viability of L929 cells in different concentration of ZnO@CuO coaxial nanofibers for 1, 2 and 3 days.**

**
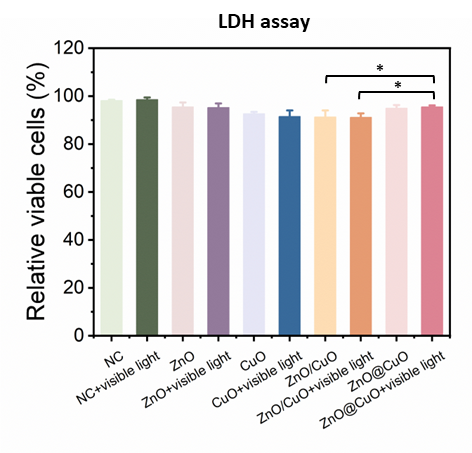
**

**Figure S7. The relative number of viable cells in different groups assessed by the LDH assay (n=3; mean ± SD; one-way ANOVA with Tukey’s post hoc test; * Significant differences of group ZnO@CuO+visible light vs. ZnO/CuO, ZnO@CuO+visible light vs. ZnO/CuO+visible light, *P* < 0.05).**

**Figure S8. Plate-coated images of methicillin-resistant *S. aureus* and *E. coli* in different groups.
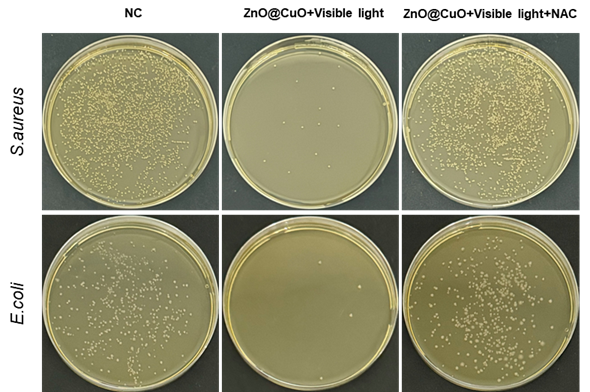
**


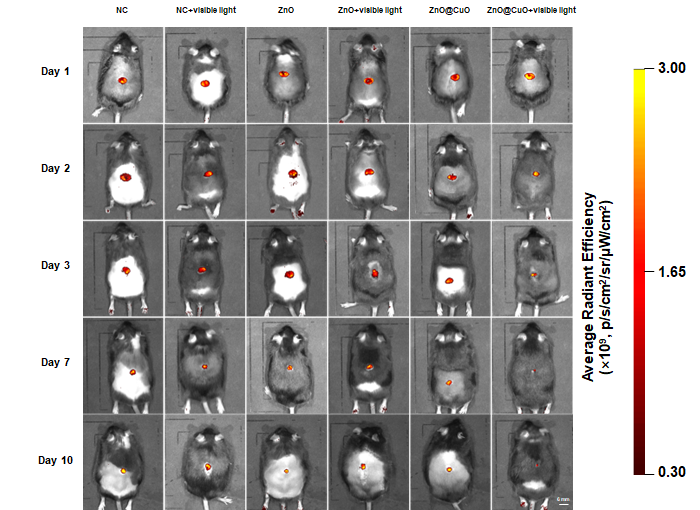


**Figure S9. *In vivo* ROS fluorescence imaging in db/db mice at different time points in different groups.**

**Figure S10. Immunohistochemical staining of 8-OHdG and TNF-α at the skin wound sites of db/db mice at 4 days. The red arrows pointed to cells with negative expression. Scale bar: 50 μm.
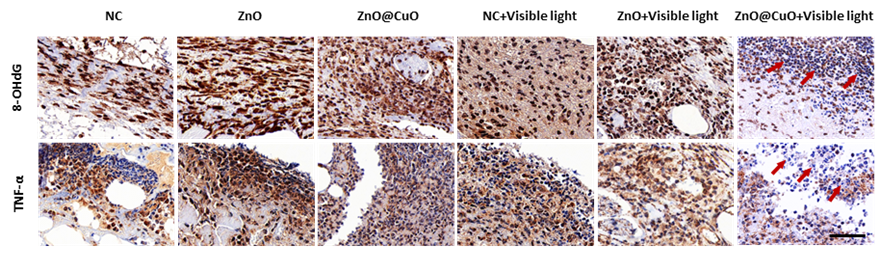
**

Supplementary references:

[1] Tong C, Zhong X, Yang Y, Liu X, Zhong G, Xiao C, Liu B, Wang W, Yang X. PB@PDA@Ag nanosystem for synergistically eradicating MRSA and accelerating diabetic wound healing assisted with laser irradiation. *Biomaterials*. **2020**, *243*, 119936.

[2] Huang S, Xu S, Hu Y, Zhao X, Chang L, Chen Z, Mei X. Preparation of NIR-responsive, ROS-generating and antibacterial black phosphorus quantum dots for promoting the MRSA-infected wound healing in diabetic rats. *Acta Biomater*. **2022**, *137*, 199.

[3] Zhao X, Chang L, Hu Y, Xu S, Liang Z, Ren X, Mei X, Chen Z. Preparation of Photocatalytic and Antibacterial MOF Nanozyme Used for Infected Diabetic Wound Healing. *ACS Appl Mater Interfaces*. **2022**, *14*, 18194.

[4] Zhu W, Liu YQ, Liu P, Cao J, Shen AG, Chu PK. Blood-Glucose-Depleting Hydrogel Dressing as an Activatable Photothermal/Chemodynamic Antibacterial Agent for Healing Diabetic Wounds. *ACS Appl Mater Interfaces*. **2023**, *15*, 24162.

[5] Li Y, Fu R, Duan Z, Zhu C, Fan D. Artificial Nonenzymatic Antioxidant MXene Nanosheet-Anchored Injectable Hydrogel as a Mild Photothermal-Controlled Oxygen Release Platform for Diabetic Wound Healing. *ACS Nano*. **2022**, *16*, 7486.

[6] Hu S, Yang Z, Zhai Q, Li D, Zhu X, He Q, Li L, Cannon RD, Wang H, Tang H, Ji P, Chen T. An All-in-One "4A Hydrogel": through First-Aid Hemostatic, Antibacterial, Antioxidant, and Angiogenic to Promoting Infected Wound Healing. *Small*. **2023**, *19*, e2207437.

[7] Chen J, Liu Y, Cheng G, Guo J, Du S, Qiu J, Wang C, Li C, Yang X, Chen T, Chen Z. Tailored Hydrogel Delivering Niobium Carbide Boosts ROS-Scavenging and Antimicrobial Activities for Diabetic Wound Healing. *Small*. **2022**, *18*, e2201300.

[8] He C, Bi S, Zhang R, Chen C, Liu R, Zhao X, Gu J, Yan B. A hyaluronic acid hydrogel as a mild photothermal antibacterial, antioxidant, and nitric oxide release platform for diabetic wound healing. *J Control Release*. **2024**, *370*, 543.

[9] Zhu S, Li M, Wang Z, Feng Q, Gao H, Li Q, Chen X, Cao X. Bioactive Glasses-Based Nanozymes Composite Macroporous Cryogel with Antioxidative, Antibacterial, and Pro-Healing Properties for Diabetic Infected Wound Repair. *Adv Healthc Mater*. **2023**, *12*, e2302073.

[10] Ma T, Zhai X, Huang Y, Zhang M, Zhao X, Du Y, Yan C. A Smart Nanoplatform with Photothermal Antibacterial Capability and Antioxidant Activity for Chronic Wound Healing. *Adv Healthc Mater*. **2021**, *10*, e2100033.

[11] He Y, Liu K, Guo S, Chang R, Zhang C, Guan F, Yao M. Multifunctional hydrogel with reactive oxygen species scavenging and photothermal antibacterial activity accelerates infected diabetic wound healing. *Acta Biomater*. **2023**, *155*, 199.

[12] He D, Liao C, Li P, Liao X, Zhang S. Multifunctional photothermally responsive hydrogel as an effective whole-process management platform to accelerate chronic diabetic wound healing. *Acta Biomater*. **2024**, *174*, 153.

[13] Zhao N, Yuan W. Antibacterial, conductive nanocomposite hydrogel based on dextran, carboxymethyl chitosan and chitosan oligosaccharide for diabetic wound therapy and health monitoring. *Int J Biol Macromol*. **2023**, *253*, 126625.

[14] Liu L, Zheng J, Li S, Deng Y, Zhao S, Tao N, Chen W, Li J, Liu YN. Nitric oxide-releasing multifunctional catechol-modified chitosan/oxidized dextran hydrogel with antibacterial, antioxidant, and pro-angiogenic properties for MRSA-infected diabetic wound healing. *Int J Biol Macromol*. **2024**, *263*, 130225.

[15] Ding Q, Sun T, Su W, Jing X, Ye B, Su Y, Zeng L, Qu Y, Yang X, Wu Y, Luo Z, Guo X. Bioinspired Multifunctional Black Phosphorus Hydrogel with Antibacterial and Antioxidant Properties: A Stepwise Countermeasure for Diabetic Skin Wound Healing. *Adv Healthc Mater*. **2022**, *11*, e2102791.

[16] Wang Z, Ou X, Guan L, Li X, Liu A, Li L, Zvyagin AV, Qu W, Yang B, Lin Q. Pomegranate-inspired multifunctional nanocomposite wound dressing for intelligent self-monitoring and promoting diabetic wound healing. *Biosens Bioelectron*. **2023**, *235*, 115386.

[17] Xu Z, Deng B, Wang X, Yu J, Xu Z, Liu P, Liu C, Cai Y, Wang F, Zong R, Chen Z, Xing H, Chen G. Nanofiber-mediated sequential photothermal antibacteria and macrophage polarization for healing MRSA-infected diabetic wounds. *J Nanobiotechnology*. **2021**, *19*, 404.

[18] Xie J, Liu G, Chen R, Wang D, Mai H, Zhong Q, Ning Y, Fu J, Tang Z, Xu Y, Li H, Lei M, Cheng H, Huang Y, Zhang Y. NIR-activated electrospun nanodetonator dressing enhances infected diabetic wound healing with combined photothermal and nitric oxide-based gas therapy. *J Nanobiotechnology*. **2024**, *22*, 232.

[19] Fu J, Wang D, Tang Z, Xu Y, Xie J, Chen R, Wang P, Zhong Q, Ning Y, Lei M, Mai H, Li H, Liu H, Wang J, Cheng H. NIR-responsive electrospun nanofiber dressing promotes diabetic-infected wound healing with programmed combined temperature-coordinated photothermal therapy. *J Nanobiotechnology*. **2024**, *22*, 384.

[20] Yu Y, Tian R, Zhao Y, Qin X, Hu L, Zou JJ, Yang YW, Tian J. Self-Assembled Corrole/Chitosan Photothermal Nanoparticles for Accelerating Infected Diabetic Wound Healing. *Adv Healthc Mater*. **2023**, *12*, e2201651.

[21] Chen S, Li A, Wang Y, Zhang Y, Liu X, Ye Z, Gao S, Xu H, Deng L, Dong A, Zhang J. Janus polyurethane sponge as an antibiofouling, antibacterial, and exudate-managing dressing for accelerated wound healing. *Acta Biomater*. **2023**, *171*, 428.

[22] Yang X, He S, Wang J, Liu Y, Ma W, Yu CY, Wei H. Hyaluronic acid-based injectable nanocomposite hydrogels with photo-thermal antibacterial properties for infected chronic diabetic wound healing. *Int J Biol Macromol*. **2023**, *242*, 124872.

[23] Wang P, Pu Y, Ren Y, Kong W, Xu L, Zhang W, Shi T, Ma J, Li S, Tan X, Chi B. Enzyme-regulated NO programmed to release from hydrogel-forming microneedles with endogenous/photodynamic synergistic antibacterial for diabetic wound healing. *Int J Biol Macromol*. **2023**, *226*, 813.

[24] Cai Y, Xu X, Wu M, Liu J, Feng J, Zhang J. Multifunctional zwitterionic microneedle dressings for accelerated healing of chronic infected wounds in diabetic rat models. *Biomater Sci*. **2023**, *11*, 2750.

[25] Fan D, Xie R, Liu X, Li H, Luo Z, Li Y, Chen F, Zeng W. A peptide-based pH-sensitive antibacterial hydrogel for healing drug-resistant biofilm-infected diabetic wounds. *J Mater Chem B*. **2024**, *12*, 5525.

[26] Huang W, Yuan H, Yang H, Tong L, Gao R, Kou X, Wang J, Ma X, Huang S, Zhu F, Chen G, Ouyang G. Photodynamic Hydrogen-Bonded Biohybrid Framework: A Photobiocatalytic Cascade Nanoreactor for Accelerating Diabetic Wound Therapy. *JACS Au*. **2022**, *2*, 2048.

[27] Wei T, Pan T, Peng X, Zhang M, Guo R, Guo Y, Mei X, Zhang Y, Qi J, Dong F, Han M, Kong F, Zou L, Li D, Zhi D, Wu W, Kong D, Zhang S, Zhang C. Janus liposozyme for the modulation of redox and immune homeostasis in infected diabetic wounds. *Nat Nanotechnol*. **2024**, *19*, 1178.

[28] Peng Y, Guo Y, Ge X, Gong Y, Wang Y, Ou Z, Luo G, Zhan R, Zhang Y. Construction of programmed time-released multifunctional hydrogel with antibacterial and anti-inflammatory properties for impaired wound healing. *J Nanobiotechnology*. **2024**, *22*, 126.

[29] Hu YW, Wang YH, Yang F, Liu DX, Lu GH, Li ST, Wei ZX, Shen X, Jiang ZD, Zhao YF, Pang Q, Song BY, Shi ZW, Shafique S, Zhou K, Chen XL, Su WM, Jian JW, Tang KQ, Liu TL, Zhu YB. Flexible Organic Photovoltaic-Powered Hydrogel Bioelectronic Dressing With Biomimetic Electrical Stimulation for Healing Infected Diabetic Wounds. *Adv Sci (Weinh)*. **2024**, *11*, e2307746.
